# Sexual assault and fatal violence against women during the Irish War of Independence, 1919–1921: Kate Maher’s murder in context

**DOI:** 10.1136/medhum-2021-012178

**Published:** 2021-11-05

**Authors:** Ciara Breathnach, Eunan O'Halpin

**Affiliations:** 1 History, University of Limerick Faculty of Arts Humanities and Social Sciences, Limerick, Ireland; 2 History, Trinity College Dublin, Dublin, Ireland

**Keywords:** history, medical humanities, medical ethics/bioethics, gender studies, law

## Abstract

At the height of the Irish War of Independence, 1919–1921, 45-year-old Kate Maher was brutally raped. She subsequently died of terrible wounds, almost certainly inflicted by drunken British soldiers. This article discusses her inadequately investigated case in the wider context of fatal violence against women and girls during years of major political instability. Ordinarily her violent death would have been subject to a coroner’s court inquiry and rigorous police investigation, but in 1920, civil inquests in much of Ireland were replaced by military courts of inquiry. With the exception of medical issues, where doctors adhered to their ethical responsibility to provide clear and concise evidence on injuries, wounds and cause of death, courts of inquiry were cursory affairs in which Crown forces effectively investigated and exonerated themselves. This article adopts a microhistory approach to Maher’s case to compare how civilian and military systems differed in their treatments of female fatalities. Despite the fact that the medical evidence unequivocally showed that the attack was of a very violent sexual nature, the two soldiers directly implicated were not charged with rape or any other sexual offence. In her case, and in those of other women who died violently while in the company of soldiers and policemen, prosecutions of the men involved resulted in acquittal by military court martial. This was so both for women portrayed as of immoral character and for others assumed to be ‘respectable’. It also reflects on the wider question of sexual violence during the Irish War of Independence, concluding that while females experienced a range of gender-determined threats and actions such as armed raids on their homes, the ‘bobbing’ of hair and other means of ‘shaming’, rape, accepted as the most serious act of sexual assault, was regarded by all combatants as beyond the pale.

## Introduction

**Figure 1:**
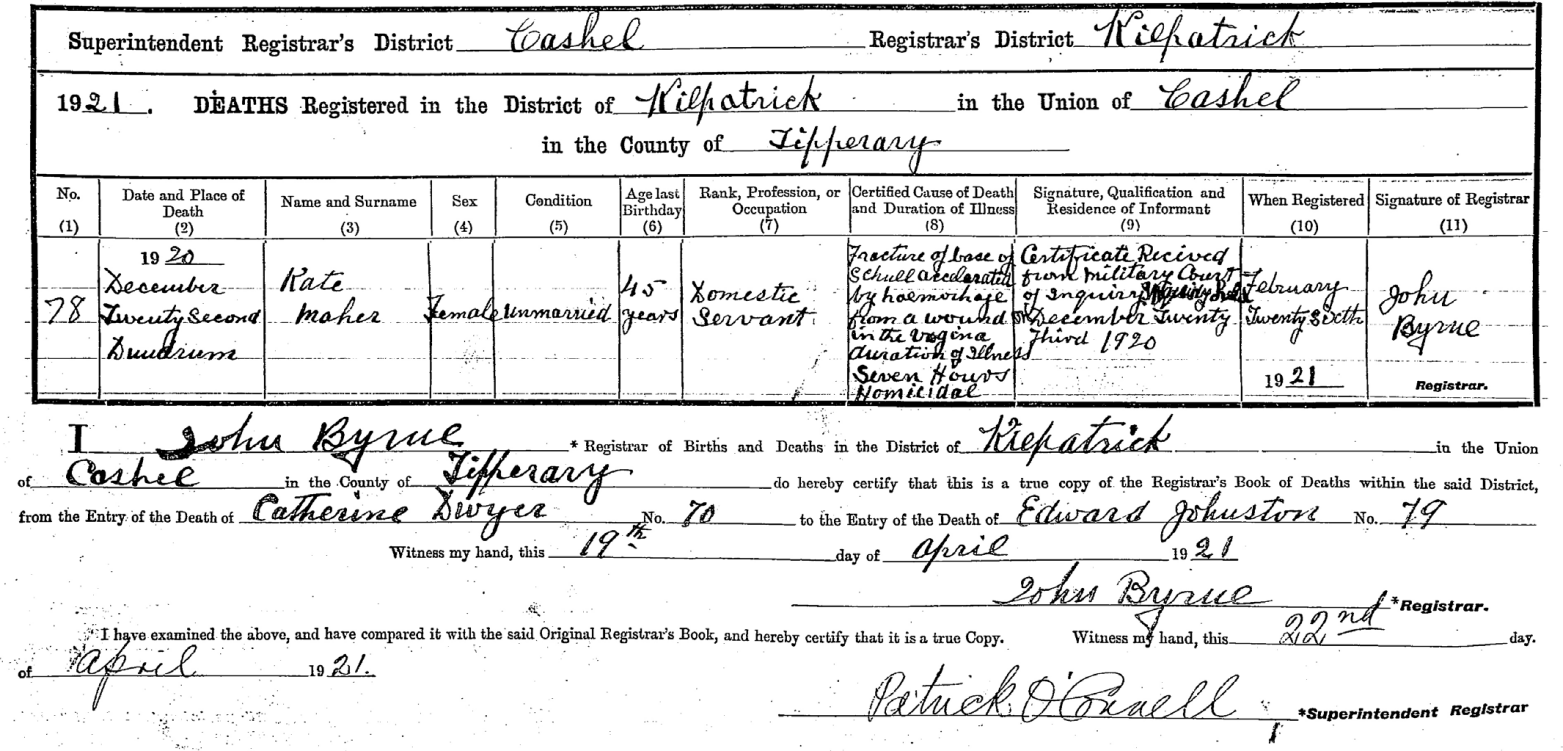
Kate Maher’s death registration.

On 26 February 1921, Dr John Byrne, registrar in the district of Kilpatrick, County Tipperary, registered the death of Kate Maher, a 45-year-old farm servant and unmarried mother, using information received from a military court of inquiry. As figure 1 shows, the cause of death, which occurred on 22 December 1920, was ‘Fracture of Base of schull [sic] accelerated by haemorrhage from a wound in the vagina duration of illness Seven Hours Homicidal’ (General Registrar Office (GRO), Maher 1920). Kate Maher had spent her last evening with British soldiers, who had taken advantage of the absence of superior officers to get drunk and cause considerable disruption in the small village of Dundrum. While investigating reports of disorderly behaviour, two ‘Black and Tans’—temporary constables of the Royal Irish Constabulary (RIC)—discovered Kate Maher lying in the yard behind Hennessy’s public house, dying from wounds to her skull and to her vagina.

Joanna Bourke has concluded from her extensive research on sexual violence that its study is difficult because of ambiguous definitions: ‘contradictory coinages and euphemisms are commonplace’ (Bourke 2007, 13). To broaden the scope of her work, Bourke crafted her own definition: ‘sexual abuse is any act called such by a participant or third party’ (Bourke 2007, 14). Her definition is important to this study because it provides a framework to detect or interpret cases that do not directly mention rape or sexual violence. An example of the prevailing restraint is provided in a circular letter of protest about British security policy addressed to British MPs by Éamon de Valera: writing as President of Dáil Eireann, he used the decorous term ‘outraging of women and girls’ to allege that Crown forces had resorted to sexual violence (Bull 5/3; Bull).

Even in its brevity, the details recorded in Kate Maher’s civil death registration entry indicates that her death followed a serious sexual assault. Using Bourke’s definition, on the evidence presented in the court of inquiry and the subsequent court martial, it is highly likely that one or more of the soldiers with whom she had been drinking attacked her. A drunken soldier was found lying unconscious nearby (TNA, n.d.).

Coleman (2015) discussed Maher’s case in the wider context of violence against women during the Irish revolution. This article supports her conclusion that Maher’s case stands out both for its ghastly circumstances and for its rarity. It is one of a small number where female deaths during the Irish revolution (1916–1921) appear to have arisen from the personal and, in most instances, sexual motives of men serving in one or other of the contending Crown and separatist forces. By Crown forces is meant the British military, the RIC including the notoriously undisciplined ‘Black and Tans’, and the Auxiliary Division (ADRIC), a mobile gendarmerie of ex-military officers who operated independently of the regular police. Leeson estimated that of 4342 such temporary recruits in 1920, 339 were Black and Tans located in the main towns and villages of County Tipperary in early 1921 (Leeson 2011, 26–7). They fortified pre-existing garrisons, such as Dundrum, which is why soldiers feature so centrally in Maher’s case. The separatist force was the Irish Volunteers, by 1920 generally known as the Irish Republican Army (IRA). There was a cross-over in membership between the IRA and the political party Sinn Féin which controlled the independence movement.

This article draws on the recently published *The Dead of the Irish Revolution*, which chronicles and analyses all known deaths arising from Irish political violence from 21 April 1916 to 31 December 1921. That work reports that Maher’s is one of just 4 per cent (98) of female deaths of 2346 arising from Irish political violence between 1917 and 1921. Only a handful of those deaths were the result of the deliberate targeting of females—just three women were definitely killed by the IRA as ‘spies’ (informers)—and in only seven cases is there hard evidence or strong suggestions of a personal or sexual motive. Six of those cases involved police or military suspects; in the seventh, the likely perpetrator was a civilian supporter of the independence movement, a connection which the RIC maintained had hampered effective investigation of the crime.

We focus here on the War of Independence era because the political circumstances gave rise to martial law, courts of inquiry were cursory affairs in which Crown forces effectively investigated and exonerated themselves. By locating the details of Maher’s vicious assault in their wider socio-political and cultural circumstances, this article offers further insights into the weak positioning of women who were othered by what Louise Ryan has termed a ‘cult of female purity’ that all too readily classified Irish women as either victims or viragos (Ryan 2000, 90). With its focus on official, if biased, sources it adds to the burgeoning field of studies of sexual violence in Ireland and builds on pioneering work by Ryan (2000)and Sandra McAvoy (2008).

Recent work has focused predominantly on testimonies from those who survived the ordeals of crimes of a sexual nature, but Maher did not live to recount her story (Byrne 2021; Clark 2020; Connolly 2021; Connolly 2020; Earner-Byrne 2015). This article focuses on the medical evidence in fatal cases of women who were not politically active during the War of Independence. It makes no claim to representations of the overall experiences women had of intimidation and the threat of physical or sexual assault, which were both insidious and widespread, and the full extent of which has yet to be determined. Instead, it contends that how rape was reported and classified by the authorities was highly sensitive to the ‘type’ of victim in question: women from the poorer classes, and particularly single mothers like Maher, and prostitutes, could expect no justice. With the exception of the evidence given by doctors, the women discussed here were quickly forgotten. Viewed as traitors for consorting with the enemy, their cases were unsuitable as evidence of British atrocity for separatist propaganda.

Rape, classified as a felony under section 48 of the Offences Against the Person Act (1861), and other forms of sexual defilement were consistently under-reported in Ireland. Consequently, as Mark Finnane has argued, ‘sexual violence is notoriously difficult to study with any confidence in the value of statistics’ (Finnane 2001, 519). No judicial statistics were returned for 1920 or 1921. But even in normal times, the record of criminal investigations of sexual assault cases was abysmal. In 1919, the last year for which civil administrative records are available under British administration, only one case involving a sexual assault was recorded in the Irish judicial statistics, and there were no prosecutions. No instances of defilement of girls under 16 or 13 were reported, although two cases dating from earlier years saw the perpetrators receive some months’ imprisonment. Only one other sexual offence was reported, that of incest, and there were no rape prosecutions. Five cases of indecent assault were successfully prosecuted, with sentences ranging from 2 to 6 months (Judicial Statistics 1919, 8, 12).

The reluctance of the civil authorities to prosecute in cases of sexual assault was compounded by the frequent unwillingness of victims to press charges. Taking cases against perpetrators of sexual assault posed a risk of further reputational damage for victims and their families, while women of the poorer classes, and prostitutes, were unlikely to be taken seriously by the police. These informal barriers to justice ensured that very few cases of sexual violence against females came before the Irish courts. One study covering the late nineteenth century indicates that of the few that did, about half resulted in convictions (Conley 1995, 811). Death following sexual assault was rarely reported in Ireland.

Isolated instances of sexual violence though not of rape involving Crown forces during the War of Independence were used for separatist propaganda purposes, as Louise Ryan has argued, ‘within gendered discourses of nationalism and colonialism’ (Ryan 2000, 74). Ryan maintains that such assaults were calculated to emasculate Irish men who could not defend their womenfolk. She emphasises violation of the domestic, female sphere through police and military raids in tactical terms (Ryan 2000, 80). Yet *The American Commission on Conditions in Ireland: Interim Report*, The American Commission (1921) which was extremely critical of the conduct of Crown forces towards the general public, nevertheless commended the British high command ‘for controlling the sexual licentiousness of its men’, concluding that there was no evidence of rape by military or police during the War of Independence (1921). There were certainly some rumours of serious sexual assault: the playwright Lady Augusta Gregory wrote that a Galway doctor told her that ‘the family of the girls violated by the Black-and-Tans wish it to be hushed up’, and ‘another case of the same sort … is to be kept quiet’ (Robinson 1946, 138). But claims of rape as distinct from other gendered brutalities, such as hair cropping and the invasion of the female domestic sphere, were not a dominant theme in Irish separatist propaganda.

Clark (2020), using Compensation (Personal Injury) Committee records, has argued that while some sexual violence was perpetrated, there is little to no evidence to support its tactical use during the Irish civil war of 1922–1993. This article agrees and argues that the same holds good during the War of Independence and during the overtly ethnic violence experienced in Northern Ireland between 1920 and 1923. The latter conflict, where Catholic nationalist communities were disproportionately the targets of Protestant loyalist and security force attacks, produced few allegations of specifically sexual violence. This challenges any assumption that newly independent Ireland’s specifically ‘Catholic ethos’ explains the absence of the ‘tactical gendered violence’. Research on widespread variations in the incidence of rape during more recent civil conflicts supports the view that more complex processes than straightforward religious conservatism underlie the presence or absence of rape (though not of less extreme forms of sexual assault and menace) (Kay Cohen 2013).

British forces were no strangers to controversies concerning the killing of civilians or to gender-based violence. The main source of guidance available to officers administering justice was the *Manual of Military Law*. As well as procedural matters and laws relating to military organisation, operations and general conduct, this carried extensive stipulations on rape, sexual assault, defilement of children, concealment of birth and related crimes which mirrored the civil law. Iteration of the range of such offences and appropriate penalties took up more space in the volume than did ‘homicide’ (Manual of Military Law 1894, 94–98, 98–100). Military cognisance of such crimes is explained partly by the need to control the conduct of the military in contact with the general civilian population, but also because of issues arising with soldiers’ families living in military quarters, both in the UK and overseas. We might note that allegations of rape against British troops reacting to nationalist disturbances in an Egyptian village in 1919 caused greater parliamentary outrage than did the action of a commander who unilaterally ordered the execution of five (male) village elders without any due process, forewarning or proper burial (Kitchen 2015, 262). Sexual assault was scarcely too delicate a subject for publicity and propaganda by the contending forces in Ireland: the Irish and international public were already well accustomed to the ‘gendered representation’ of atrocities arising from German treatment of Belgian civilians in the early months of the First World War during what was proclaimed ‘the rape of Belgium’ (Gullace 1997, 716; Kramer 2007). There was also an explicitly gendered dimension to British justifications offered for the notorious Jallinawalla Bhag massacre in Amritsar in April 1919, in which hundreds of Indian civilians died when fired on ‘without warning … they made no hostile movement’: in defending the action, much was made of an assault, physical rather than sexual, by male rioters on a British woman missionary during disturbances (Gwynn 1934, 46, 52).

## Legal procedure

The autumn of 1920 saw the military take over the administration of judicial affairs in Irish counties deemed particularly disturbed. The Defence of the Realm Act (1914) introduced what Campbell termed ‘a vast maze’ of regulations that greatly increased the power of the state, and also made provision in certain circumstances for the military to exercise certain judicial powers hitherto discharged by civil government (Campbell 1994, 10). This framework of emergency laws and regulations was replicated in the Restoration of Order in Ireland Act of August 1920 and regulations made thereunder (Campbell 1994, 27; Restoration of Order in Ireland Act 1920). Serious charges involving violence and certain political offences, normally prosecuted in civil courts, were instead adjudicated by military courts martial in what Campbell describes as a ‘progressive degradation of procedure’ (Campbell 1994, 54). Courts martial were essentially instruments for maintaining military discipline, not for dispensing justice dispassionately for the civilian population. Furthermore, most military officers involved had no legal knowledge or experience.

Coronial courts had a purely inquisitorial function and were primarily courts of record to identify the deceased and the cause of death in cases of suspicious or sudden death. According to the Coroners (Ireland) Act 1881, inquiries usually occurred within 24 hours of the discovery of a body. The first stage involved a preliminary police report and the local coroner then determined whether or not an inquest was necessary. Coroners were either legal or medical professionals. Ordinarily the coroner could call a minimum of 12 and a maximum of 23 jurors who were rate payers of over £4 and resident in the jurisdiction. Women were not permitted to serve as jurors, and the property requirement meant that those called to serve were invariably of a higher social class than the subjects of inquiry. In all cases, a medical expert, usually a local doctor with no specialist training in pathology, gave evidence. This was a normal procedure in Ireland where acrimony between ‘the priesthood of pathologists’ and general practitioners did not arise as it had in England (Burney 2000, 117). Their conclusions on cause of death were accepted by juries and were then returned as verdicts. Jurors were also permitted to add a rider, and while these had no legal standing, they often reflected local opinion and an appetite for judicial inquiry into wrongful death through the criminal courts. Maher’s suspicious death arising from a sexual assault was highly unusual by Irish standards. Rarely was such a verdict returned by a coroner’s court (military or civilian), and it was equally unusual for a registrar to record details of this nature. While verdicts were supposed to be registered verbatim as ‘information received’ from coronial inquiries, registrars, who were local dispensary doctors, often exercised their discretion and abbreviated the record. Superintendent Registrars who oversaw the process of civil registration could also suggest amendments.

Army headquarters in Dublin provided general guidance to the replacement military courts. A court of inquiry, which like a civil inquest should take place within 24 hours of death, need only be convened ‘where there is a suspicion that the death is the wilful act or the default of some other person or is self-inflicted’. Courts should consist of at least three officers, at least one of whom should be of field rank (a major or higher). Of the other members, ‘where practicable and the nature of the case requires it’, one could be an army doctor (The National Archives of the United Kingdom). Where death occurred in military hospitals, army medical officers conducted postmortems and gave evidence. But in most cases, the requirement for speed meant that the task fell on general practitioners in the locality where the death occurred. This was so irrespective of whether the victims were soldiers, police, rebels or civilians: the rudimentary postmortems on 16 of the ADRIC and RIC men killed in the celebrated—or notorious—Kilmichael ambush in November 1920 were performed by a local doctor whose own son, an RIC inspector, had been shot dead by the IRA weeks previously in another county (TNA n.d.; O’Halpin and Corráin 2020, 207, 239–41).

Courts of inquiry were usually composed of a major and two junior officers, often recently commissioned second lieutenants. They could compel witnesses to attend on pain of a fine for failure to do so, but many civilians refused to participate for political reasons or for fear of IRA retribution. Medical personnel were paid to give evidence at the daily rate of £1.1s (a guinea), and received £5.5s.0 ‘for analysis of any matter or thing of or concerning any dead body’, that is, conducting a postmortem. Proceedings were generally open to the public, but all or part of an inquiry could be held in camera to protect the identities of witnesses (The National Archives of the United Kingdom).

These new responsibilities placed an unwelcome additional burden on the army in disturbed areas. Douglas Wimberley described how as his battalion’s adjutant he had to conduct civil registrations of births, deaths and marriages as well as ‘all the inquests, of which … there were certainly plenty’. Describing many inquests as ‘rather macabre’, he recalled one inquiry where an infant had been asphyxiated with a rag (Acc 6119, Wemberley n.d., 152).

Political unrest in Ireland meant that Kate Maher’s death was not investigated by the civil authorities in the normal fashion. Ireland was in uproar. From January 1920, in the face of growing separatist violence, the British government resolved to meet fire with fire. The army in Ireland was strengthened, although the chief of the imperial general staff noted sardonically that most soldiers in Ireland were ‘raw untrained children’ (cited in O’Halpin 2020, 53–4). RIC numbers were bolstered by thousands of British ex-servicemen recruited as temporary policemen, the ‘Black and Tans’. These received little training, and they were encouraged to treat the general population as hostile. They were later joined by the ADRIC, composed of ex-officers, who acted independently mainly in a counter-insurgency role. These developments, and the IRA’s successful efforts to isolate the police, meant that the RIC, hitherto a highly successful and robust force recruited from the mainly rural Irish population which it policed, was progressively withdrawn from the rural hinterland and concentrated in towns and villages such as Dundrum. This reduced the force’s interaction with the general public, and its general effectiveness as a body which, although armed, had usually policed by consent and had operated within the law.

It was not only the Irish separatist movement and the general population who complained about the aggressive and brutal conduct of these new recruits. Major General Douglas Wimberley, who served in Ireland in 1920–1921, maintained that many of the Black and Tans and ADRIC ‘were undoubtedly no more or less than real ‘thugs’. They were totally undisciplined … and members of this curious force undoubtedly committed many atrocities’ (Wimberley).

## Maher’s case in context

The 1916 Rising and its aftermath saw the deaths of 504 people, including 281 civilians (56% of all fatalities), of whom 55 (20% of civilians, 11% of all deaths) were female. These female fatalities arose entirely because most of the fighting took place in the densely populated centre of Dublin city. O’Halpin and Ó Corráin found no personal, gender or sexual motive in any female death. Between 1917 and 1921, there were 98 female deaths (4 per cent) within the 2346 recorded as linked to political violence. The great majority were clearly unintended, the women and girls involved dying either in traffic accidents with official vehicles (15 per cent), killed as bystanders during engagements between Crown forces and the IRA, or casualties of indiscriminate firing during intercommunal violence in Belfast city (where 27 per cent of all female fatalities arose).

It is clear that for all parties involved in political killing on the island of Ireland—IRA, civilians, RIC, the British military, the Ulster Special Constabulary (an entirely partisan body, in effect an autonomous Protestant militia rather than a subordinate arm of the RIC), and loyalist groups—females were not regarded as legitimate targets. This is reflected in the IRA’s difficulty in dealing with women they regarded as spies or informers, usually because these kept company with policemen or soldiers. In November 1920, IRA headquarters ‘General Order No 13’ on ‘Women Spies’ (Foulkes papers, document dated 17 November 1920) King’s College London instructed units that any woman convicted as a spy should be ‘ordered to leave the country within 7 days’ unless she was Irish: ‘it is not proposed to deport Irishwomen, it being hoped that the bringing of publicity on the actions of such will neutralise them’. The problem persisted, as one commander explained: ‘I have one notoriously bad case in which the girl concerned has defied the Volunteers when she was warned & another of a girl who has applied … for a job as a female searcher (working for the Crown forces)’ (University College Dublin, Mulcahy Papers, P7/A/17). Elsewhere, ‘destruction of property’ was approved as a sanction against a persistent informer. By contrast, a male civilian suspected of informing might be killed on the flimsiest of evidence: one in five of all civilians (919) who died in the conflict were male spies, one just 17 years old (Mulcahy Papers, P7/A/17).

### The killing of women for political reasons

We know of no cases where Crown forces deliberately killed a woman because of her imputed political allegiances or revolutionary activities. We have identified only three cases where the IRA definitely targeted and killed females as alleged spies, out of a total of 190 civilians so killed. In each case the action caused severe embarrassment.

The three women concerned were Mary Lindsay (11 March 1921) and Brigid Noble (15 March 1921) in Cork, and Kate Carroll (17 April 1921) in Monaghan. The IRA units responsible maintained that the women had passed information to Crown forces; in each case, the local IRA’s actions and evasive explanations raised grave doubts in headquarters (Bielenberg 2020). Long after Mrs Lindsay had been killed, the Cork IRA assured headquarters in Dublin that she was alive and well. Kate Carroll’s killing was such an embarrassment that the revolutionary government’s director of publicity advised that the IRA should either deny responsibility, or state that her execution had been unauthorised and that those responsible had been disciplined. Brigid Noble’s fate caused less difficulty initially, because details only emerged months after the Truce of 9 July 1921. The wife of an English fisherman, she had made a statement to the RIC identifying men who had ‘bobbed’ her hair in a punishment attack. When the IRA GHQ asked about her death, the local IRA implied that she had been on intimate terms with various RIC men (O’Halpin and Corráin 2020, 341–2). There may well also have been an element of moral policing in the killing of Nellie Carey (19 March 1921), who was shot while out walking with two soldiers. The local IRA claimed her killing was accidental, but she had previously been warned not to associate with the military (TNA).

**Figure 2:**
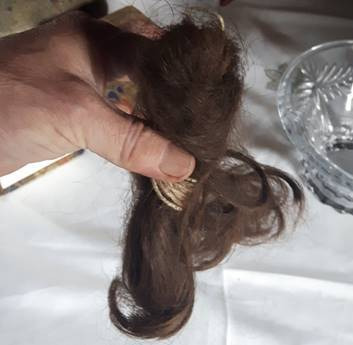
Photograph of a crudely shorn brown plait. Source: Photograph taken by Eunan O’Halpin in County Carlow, 2017, with permission to re-use.

Crown forces had comparable difficulties in dealing with women whom they believed involved in the separatist campaign, despite the fact that, as Margaret Ward’s work has shown, members of Cumann na mBan (the Irishwomen’s Council), an exclusively female separatist body structured on military lines, were highly-organised and very active (Ward 1983). Both sides often resorted to physical intimidation of women, including the forcible bobbing of hair, a mark of shame rather than an outright permanent mutilation. Mary McAuliffe and Justin Dolan Stover have described how domestic spaces were regularly violated to deliberately terrorise women (Dolan Stover 2017; McAuliffe 2020). This campaign of intimidation was widespread. Peg Broderick, Section Commander, Cumann na mBan, Galway described how in 1918 she was ‘very well known to different officers’ in Gort, Tuam, Athenry and Connemara because of her activism. In 1920, her family home was the target of an arson attempt; shortly afterwards, she was taken half-clad from her home and assaulted by Black and Tans: ‘I thought at first they were going to shoot me but they took me out … “What wonderful curls you’ve got”, and then proceeded to cut-off all my hair to the scalp with very blunt scissors’ (Military Archives of Ireland and Bureau of Military History, 4). In a statement circulated in Britain as ‘The War on Women in Ireland’, the prominent activist Madge Daly described how in October 1920 her sister ‘was knocked down, dragged on her back by the hair out of the house … her assailants cut-off her hair and slashed her hand with a razor from the back to the palm, severing an artery’ (Bourne, 5/3; Westminster Diocesan Archives). This type of violation was also employed by the IRA. The family of the celebrated executed Volunteer Kevin Barry still hold a plait (see figure 2), belonging to an anonymous Carlow woman or girl, which they say was the result of such a bobbing by the local IRA battalion of which Kevin’s brother Mick was a senior officer (O’Halpin 2020, 167–8). Such grim trophies are a reminder of the significance of the act.

*The Dead of the Irish Revolution* identified just two cases where rape or attempted rape was definitely involved in deaths. An alleged rapist, ex-soldier Michael Joseph Murray, was shot by an off-duty soldier in Cork City who heard Margaret O’Sullivan’s cries for help at night and confronted him, unintentionally shooting him. The girl’s clear evidence was that Murray ‘knocked me down & bit my cheek’ and was on top of her when the soldier intervened. So familiar was the practice of bobbing that the soldier testified that when he ‘first saw something on the ground and heard screams I thought a girl was having her hair cut off. But when I got nearer I could see that a civilian was trying to molest a girl’. A court of inquiry found that Murray was ‘attempting to rape one Margaret O’Sullivan an unmarried girl against her will’ (TNA, WO 35/155B/32). The unnamed soldier who inadvertently shot him was later acquitted of manslaughter by a court martial (O’Halpin and Corráin 2020, 336). The second case, the horrific rape and murder of Kate Maher, is discussed in detail below.

### The killing by combatants of women for personal reasons

Seven instances have been identified between 1917 and 1921 where women died through the actions of combatants—or in one case at the hands of a person held to be a strong supporter of the independence movement—where there are indications that personal motivations were involved. In all these cases, investigations were clearly affected by the disturbed condition of the country. Furthermore, in six of the seven cases (see table 1) where men were charged with the murder or manslaughter of women, justice was administered by military courts established under the Restoration of Order in Ireland Act in August 1920 (Restoration of Order in Ireland Act 1920). Such courts, designed for internal military disciplinary purposes and composed of officers most of whom had no legal training and experience, were inherently unsatisfactory in dealing with cases which were essentially civilian in character (Townshend 2014, 151–3).

**Table 1:** Women probably killed for personal reasons

Name	Date	Inquiry	Court martial or civil trial	Conviction
Elizabeth Carberry	27 October 1920	Coroner’s	Court martial	No
Mary Maher	16 December 1920	Military 21 December 1920	Unclear	No
Kate Maher	21 December 1920	Military	Court martial	No
Sarah Fitzpatrick	3 February 1921	Military	Neither	No
Mary Fahey	19 May 1921	None found	Civil trial	No
Anne Dixon	24 May 1921	Military 26 May 1921	Court martial	No
Kathleen Kelleher	3 July 1921	Military 4 July 1921	Civil trial	No

Kate Maher’s is one of three cases where women died at the hands of off-duty soldiers. The second is that of the elderly shopkeeper, Mary Maher, also of Tipperary, whose death from a blow to the head apparently inflicted by a private of the Northamptonshire Regiment seems to have occurred in a robbery which went wrong. A Private O’Brien was returned for trial by court martial, but the outcome is unknown (TNA, WO 35/155B/5). The third such case, that of Elizabeth ‘Bessie’ Carberry, is somewhat better documented. She was found suffocated in a Dublin city centre laneway on 27 October 1920 after neighbours heard a prolonged argument between her and a soldier with whom she had apparently been drinking. A 30-year-old single woman under five feet in height, she had recent convictions for drunkenness and soliciting (National Archives of Ireland, Mountjoy Prison General Register Female, 1918, 1/44/15). The experienced Dublin City Coroner Dr Louis A. Byrne, no stranger to controversial inquests, ordered that two medical witnesses should conduct postmortems independently. They noted extensive bruises to her jaws and a ‘severe bruise’ behind her left ear and another on her right thigh. As her larynx was undamaged, both doctors surmised she had not been choked or strangled: ‘The medical evidence was that death was due to suffocation and that the suffocation resulted from the pressure of a hand across the mouth’ (*Wicklow People*, October 30, 1920, *Dublin Evening Telegraph*, October 28, 1920). A policeman stated that Bessie Carberry’s ‘legs were doubled up under her, and her clothing was disarranged, indicating a struggle’ (*Freeman’s Journal*, October 28, 1920; *Irish Independent*, 17 January 1921; O’Halpin and Corráin 2020, 203). Police also found a regimental cap badge at the scene. The coroner directed the jury to return a verdict of death by suffocation, and of murder ‘against a soldier unknown’ (GRO Carberry, 29 October 1920). Lance Corporal Albert Hadley had been arrested nearby by a military curfew patrol: his cap badge was missing, and the knees of his trousers were wet as though he had been kneeling. In February 1921, by which time Dublin was under martial law, Hadley was court martialled for murder, or in the alternative manslaughter. Despite the medical and police evidence, no charge of sexual assault was laid. The case was quickly dismissed on the grounds of insufficient evidence, after it was shown that a second soldier from Hadley’s unit had also lost a cap badge in Dublin that night (TNA, WO35/93/part B; *Wicklow People*, 30 October 1920; *Dublin Evening Telegraph*, 28 October 1920; O’Halpin and Corráin 2020, 23).

We should also consider three cases in which young women were killed by the weapons of policemen with whom they were personally involved. The policemen involved were temporary recruits—two Black and Tans, and one ADRIC.

Sarah Fitzpatrick, a Scottish war widow who had come to Dublin for an engagement as a chorus line dancer in a pantomime, apparently shot herself, perhaps by accident, as she was being escorted home by her ex-boyfriend, ADRIC District Inspector ‘Tiny’ Purchase. They had both been drinking, although he was on duty. Purchase maintained to a court of inquiry that Sarah Fitzpatrick often toyed with his weapon, and that it had discharged accidentally as she held it just outside her home. A doctor testified that Sarah Fitzpatrick’s death was ‘due to shock and haemorrhage from wounds in the heart’, but was unable to form an opinion as to whether these were self-inflicted. One witness testified that they had all been drinking together that night in pubs near their home. She described ‘Connie’ as jolly, but stated that ‘On one occasion she told me she that loved Tiny, and that if anything came between them she would put a bullet in him first and then in herself afterwards’. That conversation was overheard by two other co-residents. One of these also testified that another woman had claimed that she saw Purchase shoot Fitzpatrick, but dismissed this story as that woman was ‘under the influence of drink, and I had an idea what class of girl she was’, a clear imputation of immoral character. Although the medical witness said he was unable to form an opinion as to whether her wounds were self-inflicted, the court returned a verdict of suicide, and the death was registered as ‘suicidal’ from the information received from the military court of inquiry (GRO Fitzpatrick, 5 February 1921). The inadequacy of proceedings was noted by the military authorities: the general officer commanding Dublin district wrote that ‘A case like this should have had a legal man on it. I do not think it is all satisfactory. Draw attention … to it & say that it shows in my opinion great slackness & want of supervision on the part of those responsible for the men of this [ADRIC] Coy [company]’ (TNA, WO 35/149B/15). There was a ‘large attendance of friends and theatre workers’ at Sarah Fitzpatrick’s funeral in Dublin, probably an indication of her perceived respectability as well as of general sympathy (*Freeman’s Journal* and *Irish Independent*, February 9, 1921).

The second case occurred in Clones, County Monaghan. Twenty-year-old milliner Anne Dixon was killed by her ex-boyfriend, a Black and Tan who had called to her workplace. Investigation showed that he had devised a pretext for being in Clones that day and for carrying his weapon while off-duty, that his letters to her displayed violent jealousy, and that he had earlier threatened her with his gun. Despite this uncontested evidence, a court martial accepted his defence that his revolver had gone off accidentally as he removed it from his pocket. Her last words were, reportedly, ‘Oh, my heart’s drops’ (TNA, WO35/130/20; O’Halpin and Corráin 2020, 442–3).

There are similarities between Dixon’s and the third case. In Dublin, just a week before the Truce of July 11, 1921 which ended the Anglo-Irish conflict, 17-year-old embroiderer Kathleen Kelleher was mortally wounded while sitting under a tree in the Phoenix Park when her companion, a newly recruited Black and Tan who had only known her a week, shot her in the head while removing his pistol from his hip pocket. Although some semblance of a process occurred in the aftermath, Major General GF Boyd, in command of the Dublin military district, was of the opinion that ‘In view of the abnormal conditions in this country, and the necessity for carrying a loaded revolver, and also in view of the opinion of the court (of inquiry), I suggest’ that the constable ‘should not be tried’. Nevertheless, on the directions of the general officer commanding in Ireland, the constable was charged with murder, later reduced to manslaughter, before a civil court but was quickly acquitted (TNA, WO 35/161b). An IRA veteran later claimed that Kathleen Kelleher was murdered by Crown forces because she had an IRA boyfriend, but there is no other evidence to support this theory: it was not aired publicly at the time by her family or by the separatist movement, although it would have been reasonably safe to do so in the circumstances of the Truce (O’Halpin and Corráin 2020, 291–2, 442–3, and 504–5).

Deaths at the hands of combatants are of course only a subset of violent deaths suffered by females in Ireland in those years. The unsettled conditions in Ireland plainly hindered effective investigation of crimes against women. In 1919, the IRA had embarked on a systematic campaign to break the intimate links between the RIC and the general public. This rendered them far less effective in both the prevention and investigation of crime. This is illustrated in the case of the heavily pregnant Mary Fahey, a single woman whose skull was beaten in shortly after she was seen arguing with a young neighbour whom she accused of being the father of her expected child. The RIC said they could not mount an efficient investigation because the obvious suspect was a Sinn Féin supporter. It was left to Mary Fahey’s family to identify and search the scene of the crime and discover key evidence. A bloodstained shirt-cuff seized by the RIC from the suspect’s home was sent for analysis to RIC headquarters, but was mislaid and could not be produced in court. This led inevitably to acquittal by a civil court (O’Halpin and Corráin 2020, 435). As in the case of Kate Maher, where investigation had been in the hands of poorly trained, inexperienced temporary policemen, Fahey’s death illustrates how the unsettled conditions affected the administration of justice.

## Kate Maher’s inquest

Tipperary was one of five Irish counties in which martial law was declared on 10 December 1920, so both the court of inquiry into Kate Maher’s death and the trial of her alleged murderer were in military hands (Jeffery 1984, 87). It is a matter of conjecture whether a more thorough investigation and a more energetic prosecution might have been mounted had proceedings been the responsibility of the civil authorities.

The one aspect of Kate Maher’s fate, which did receive adequate exploration, was forensic. The medical evidence at the court of inquiry and later at the court martial of her alleged killer was, while primitive by today’s standards, shocking and unequivocal. Even in the fraught conditions of 1920, where no one could have felt safe from retribution by one group of combatants or another, medical doctors performed their civic duty in accordance with their professional ethics. Kate Maher’s ghastly wounds thus entered the record and the public domain.

Dr P.T. Morrissey was a physician/surgeon who had occupied the part-time position of district coroner in the southern region of County Tipperary since 1889 (Wellcome Trust; BPP, Local government (Ireland) officials). He had conducted the inquests arising from the Soloheadbeg killings of two RIC men in January 1919, which marked the beginning of the War of Independence (*Nenagh News*, 25 January 1919). The resulting inquests had three independent medical witnesses and a jury (*Larne Times,* 1 February 1919). In contrast to the medically oriented inquests which Morrissey had overseen, in military courts of inquiry a President and two members heard evidence and made recommendations arising from their findings. In Maher’s case, a court met on 23 December 1920 under Major A.H. Bell. Presumably due to the Christmas holiday, the court did not return a verdict until 6 January 1921, whereas courts of inquiries, like coroners’ inquests, normally sat within 24 hours of the discovery of the body and issued their verdicts within hours (TNA, WO 35/155B/4). In all, seven witnesses were called. In order, they were Nora Hennessey, publican; Dr Daniel McCormick, local dispensary doctor; Patrick Hennessey, publican; RIC constables Charles Honour and Robert Joynson (TNA, HO 184/161; TNA, HO 184/62; TNA, HO 184/37; TNA); TNA, HO 184/62; TNA, HO 184/161 and privates Thomas Bennett and Private Joseph Brown, both from the Lincolnshire Regiment.

The body was formally identified by publican Nora Hennessy. She stated that Maher was an unmarried mother who worked as a servant in a nearby townland for a farmer named Matthew Ryan. We can assume that Maher and her child lodged there: in the 1911 census, Ryan was recorded as housing three lodgers, and it was customary for general servants to have room and board as part of their remuneration (Ryan Census Return 1911). Kate Maher was atypical of Irish ‘farm servants’, whom Richard Breen argues were usually under 30 years of age and usually moved on, but ‘lifetime’ servanthood was not unknown (Breen 1983, 94). Her surname is very common in County Tipperary, but she is probably the Kate Maher who gave birth to a girl named Johanna at Cashel workhouse in February 1910, no father being named in the register of births (GRO Johanna Maher, 2 April 1910). Maternity services were rudimentary outside metropolitan areas, and poor expectant mothers routinely turned to the local ‘Union’ hospital for lying-in purposes. Women like Kate Maher left faint impressions on the historical record, and indeed, it is likely that the birth of her child was only recorded as required by law because it took place in an institution.

Constables Stanley Charles Honour and Robert Joynson, ‘Black and Tans’ stationed in Dundrum, respectively, for 9 months and for just 3 weeks, were out on patrol on the ill-fated night. At about 23:00, they met a Miss Price, who complained about troops attempting to steal her chickens and generally acting in a drunk and disorderly manner in the village. At that point, according to Joynson’s testimony to the military court of inquiry, they found ‘about seven men of the Lincolnshire regiment’ and Kate Maher outside a public house. They ushered the soldiers back to barracks, but discovered on arrival that a few men were missing. They went back to the village, found two men at the back of Mrs Furlong’s public house, and escorted them back to the barracks. One soldier was still missing, and they went back again to find him. When Joynson returned to the village about 50 min later, he heard groans from behind a wall. He discovered Kate Maher lying unconscious and bleeding from her ‘head and her womb’, and he testified euphemistically that ‘her clothes were very much disturbed’ (a similar euphemism to that used to describe Bessie Carberry’s condition). Private Bennet was found a few paces away in such an inebriated state that he was unable to stand or walk. Honour picked him up while Joynson went to Hennessey’s public house to get brandy to revive Maher. The Hennesseys agreed to allow Maher to be brought to their public house and while she was being conveyed, Joynson stated that two armed soldiers came to retrieve their missing comrade, Private Bennet. Private Joseph Brown, on-guard duty that night, stated in evidence that there was no blood on Bennett when he was found.

Private Bennett was permitted to cross-examine the policemen during the inquiry in lieu of inquest, and took the opportunity to clarify points that would later work to his advantage in court martial proceedings. He asked if there was ‘anything to indicate that I had any physical or sexual connection with the deceased woman’ and asked the witnesses to confirm his high level of intoxication. It is because of this line of questioning that Maher’s case also conforms to the criteria that Joanna Bourke (2007) identifies in her definition of rape. Joynson replied that Bennet was indeed ‘in a drunken stupor’ and that there was no evidence that he had assaulted Maher. In a typical effort to discredit the victim, Constable Honour confirmed in answer to Bennett that she was known locally to be a woman ‘of dissolute habits’. Bennett also elicited the fact that the yard where Kate was found was large and had multiple exits.

On closer examination by the court, the gross negligence of the constables and their sheer lack of experience of crime scene investigation was revealed: they did not check to see if Bennett’s clothes had blood splatters, nor did they search for the weapon used to assault Kate Maher. They did, however, state that Bennett’s hands had no trace of blood on them.

Dr Daniel McCormick, recorded as a ‘civic practitioner’ (dispensary doctor), told the inquest how he had responded to a call from Nora Hennessy at 01:30 on December 22 to tend to the seriously injured Kate Maher. He found her bleeding from the eyes and nose as a result of a ‘severe contusion on the left side of her head’ which he contended ‘pointed to a fracture at the base of her skull’. She was ‘bleeding profusely’ from laceration of her vagina, and he believed all her injuries were caused by a blunt instrument.

The inquest concluded that Kate Maher had died from ‘fracture of the base of the skull accelerated by haemorrhage from a wound of [the] vagina’ sustained between the hours of 23:30 December 22 and 01:30 on December 23, from which she died 7 hours later. It decreed that the wounds were inflicted by some person or persons unknown and that they were guilty of manslaughter.

The evidence and verdict of the military court of inquiry was sent for review to Lt. Col. R. Wilson, commanding officer of troops in Tipperary, who concurred with the findings: ‘There is no direct evidence to connect … Bennett or any other soldier with this woman’s death except that he was found near her and others were seen in her company’. Further notes were added to the file in February pointing to the dishonourable behaviour of the regiment. The first dated February 15 noted, ‘This is not at all satisfactory. I have little doubt but that the woman was ill-treated by the troops and death resulted’. Another annotation dated February 16 described it as ‘a discreditable affair’ (TNA, WO 35/155B/4).

In the aftermath, local weekly newspapers carried variations of the following account: ‘Brutal Deed’, ‘A woman of the servant class, named Kate Maher, aged 55 was found with her skull beaten in, at the back of a house in the village of Dundrum, Co. Tipperary, on Tuesday night. She is said to have been alive when discovered and to have succumbed shortly afterwards’ (*Skibbereen Eagle,* December 25, 1920). An abridged version had appeared previously in the daily *Irish Independent* (24 December 1920). The Cork city daily *Evening Echo* confirmed that ‘A military inquiry will be held’ (*Evening Echo*, December 28, 1920; *Nenagh Guardian,* January 1, 1921). The newspapers did not carry details of the wounds inflicted. Instead the following wording was used: ‘A medical man said the woman’s body presented the appearance of considerable violence having been used.’

## The court martial

So inexperienced were the military in Irish civilian judicial procedures that R Wilson, Lieutenant Colonel, Commanding 1st Bn the Lincolnshire Regiment had to write on 2 March 1921 to the HQ of the 16th Infantry Battalion in Fermoy to seek advice. Privates Bennett and Capes were arrested in connection with Maher’s death and, when asked by a Dublin Castle official, why the case was delayed, the Major General Commanding 6th Division responded that it was because RIC witnesses ‘were scattered in different stations’. There was a litany of failures on the part of the Crown forces.

Murders were notoriously hard to prosecute in Ireland even ordinary times. Unless the case was particularly heinous, witnesses were slow to give evidence, and thus very few such cases came before the courts (Breathnach 2020). Use of a charge of manslaughter, which carried a punishment of penal servitude, offered better prospects of conviction by a jury. But evidential flaws notwithstanding, Private Thomas Bennett was charged with murder. Newspapers reported on proceedings in some detail, though with recourse to euphemism: the word ‘vagina’ did not appear in the *Irish Examiner*’s account of the court martial, held in Cork on 16 July 1921. Kate Maher had a wound on the right side of her skull and a ‘lacerated wound on another part of her body from which a certain amount of haemorrhage had come’ (*Irish Examiner*, July 18, 1921). None of the evidence presented pointed to Bennett, who was so ‘incapably drunk’ that medical witness believed he could not have committed the attack. He had been drinking alone and had consumed a combination of beer, stout and whiskey. He testified that he had noticed Kate Maher drinking with other soldiers before she went off with them, but said he had nothing to do with that group. He was so drunk when he left to return to the barracks that he passed out, and was later found comatose near where Kate Maher lay bleeding to death. His counsel raised the question of why another suspected soldier, a Private Capes, had been suddenly drafted out to serve in Russia. Capes had initially been arrested but, because none of the eye witnesses could identify him, had been released without charge.

The solitary charge which Bennett faced was murder, which unlike rape carried a maximum penalty of death. Third parties described the sexual assault which Maher endured in euphemistic terms. Constable Joynson mentioned her disturbed clothes, and that she was bleeding from her womb. That she had been sexually assaulted was clear from Dr McCormick’s evidence. The specific question which Bennett had raised during the court of inquiry about ‘sexual connections’ was not explored.

The level of medical detail presented in the court of inquiry and court martial is insufficient to determine whether what Kate Maher endured at the hands of one or more soldiers constituted rape. The criminal law required that there had to be proof of penetration, as was also stipulated in the *Manual of Military Law* which guided courts martial. The presence of semen was not considered necessary: ‘the slightest penetration will be sufficient’.

The use of objects to penetrate a female did not then constitute rape in Irish law. It is clear Kate Maher was so assaulted. The doctor’s evidence was that she had been violently assaulted by the insertion of a blunt instrument into her vagina which had caused her to bleed profusely. It is not possible now to determine if she was also raped, as Willemijn Ruberg has done in comparable Dutch cases of sexual assault (Ruberg 2013).

Historically, court reporting of heinous crimes was necessarily selective, Irish newspapers generally avoided graphic descriptions of wounds or injuries of an intimate or sexual nature disclosed during court proceedings, presumably out of deference to public sensibilities. Reporting on Private Bennett’s court martial, one newspaper spoke of Kate Maher’s skull fracture and what was delicately termed ‘a lacerated wound on another part of her body’ (*Freeman’s Journal*, July 18, 1921). Given the heightened state of unrest and the danger of official or unofficial military reprisals against newspapers, detailed coverage carried risk.

## Conclusions

Kate Maher’s terrible death at the end of a difficult life was one of only a handful during the War of Independence where women were killed by men in circumstances suggesting that personal or sexual factors were involved. While the initial casual and inept investigation of her death was partly attributable to the lack of training of the temporary policemen involved, the manner in which both the military court of inquiry and the ensuing court martial were conducted demonstrates the systemic unsuitability of the military system of justice, designed essentially to maintain discipline within the armed forces, for dealing with cases involving civilians.

This article supports Louise Ryan’s contention that both Crown and separatist forces were culpable in varieties of sexual harassment and assault on women, but that there is no evidence that the most extreme forms of sexual violence—rape and sexually motivated murder—were widespread, still less that they were accepted forms of coercion. That the deaths at soldiers’ hands of Kate Maher and Elizabeth ‘Bessie’ Carberry were not seized on by the separatist movement for propaganda purposes undoubtedly reflects their low social standing and presumed moral failings. The IRA themselves routinely used gendered forms of violence against women who associated with police and soldiers. Yet the rapid acquittal of the Black and Tan policemen who killed the ‘respectable’ Anne Dixon and Kathleen Kelleher suggests that a female victim’s perceived social status alone was not enough to secure thorough investigation and prosecution. The disturbed conditions gave their would-be suitors a ready-made excuse for carrying weapons while off-duty, and military courts appear generally to have provided a sympathetic environment for soldiers or policemen accused of killing civilians than would a civil court presided over by an experienced judge.

Kate Maher’s case stands apart from all the others because of the unequivocal evidence presented of extreme sexual violence. Although her body bore evidence of at least an attempt to rape, Bessie Carberry’s death from suffocation was somehow more mundane and less indelicate for the newspapers. The failed prosecution of her alleged killer may be partially explained by the disturbed conditions of the time, but the fact that the case was heard by a military court composed of officers the majority of whom had no legal expertise was also significant. It is also likely that, as with Kate Maher, Bessie Carberry’s social standing and implicit low morals were contributory factors to the inadequate pursuit of justice for her. The best that can be said of military courts of inquiries in lieu of inquest is that they served the purpose of identification of the deceased, and determination of the cause of death. It is also useful that their records, along with those of the equally unsatisfactory court martials, were so carefully collated and well preserved by the military authorities, quite unlike most Irish civil court records. But researchers should not be deceived by their relative orderliness and completeness: what they reflect above all is not the procedural exactitude of military administration of the law, but its patent inadequacy in cases involving civilian deaths.

The exception to the general failings of military justice in serious civil cases arises in the unequivocal medical evidence supplied both to courts of inquiry and to courts martial. In all the cases discussed here which came to trial, doctors presented their findings, including those pointing towards sexual motives, in as clear and unambiguous a manner as though they were before a civil court in quiet times, and without reference to the victims’ backgrounds or imputed moral character. In that respect, though in no other, the unmarried mother Kate Maher, and sex worker Bessie Carberry, were fairly served as were more ‘respectable’ young women who died at the hands of their police or military companions’.

10.1136/medhum-2021-012178.supp1 Supplementary data List of durable URLs for civil registration of deaths.

## Data Availability

Data sharing not applicable as no datasets generated and/or analysed for this study. This article is based on archival research, where digitised and freely available URLs are provided.

## Notes

1. *Freeman's Journal*, February 9, 1921.

2. *Irish Independent,* February 9, 1921
