# Supplementary material for: Sexual assault and fatal violence against women during the Irish War of Independence, 1919–1921: Kate Maher’s murder in context
Source: Med Humanit. 2021 Nov 5;48(1):94–103. doi: 10.1136/medhum-2021-012178 (PMC8867259; doi:10.1136/medhum-2021-012178)
Supplement: Supplementary data [file medhum-2021-012178supp001.pdf]

Archival appendix.

General Register Office Records

https://civilrecords.irishgenealogy.ie/churchrecords/images/deaths_returns/deaths_1921/05103/4402237.pdf accessed 10 December 2020. Death registration Kate Maher.

https://civilrecords.irishgenealogy.ie/churchrecords/images/deaths_returns/deaths_1921/05105/4402910.pdf Accessed 8 December 1920. Death registration Sarah Fitzpatrick.

https://civilrecords.irishgenealogy.ie/churchrecords/images/deaths_returns/deaths_1920/05112/4405503.pdf Accessed 8 December 2020. Death registration Elizabeth Carberry.

https://civilrecords.irishgenealogy.ie/churchrecords/images/birth_returns/births_1910/01569/1633076.pdf Birth registration of Johanna Maher 2 April 1910.

Matthew Ryan Dundrum, Residents of a house 10 in Dundrum (Kilpatrick, Tipperary)
http://www.census.nationalarchives.ie/reels/nai003294763/
